# The transition towards a circular economy. A framework for SMEs

**DOI:** 10.1007/s10997-022-09653-6

**Published:** 2022-09-11

**Authors:** Francesca Gennari

**Affiliations:** Department of Economics and Management, University of Brescia, Brescia, Italy

**Keywords:** Circular economy, SMEs, Transition management theory, Sustainability management

## Abstract

Small and Medium Enterprises (SMEs) contribute significantly to the European GDP and play a pivotal role in the ecological transition from a linear to a circular economy (CE). According to transition management theory, which emphasizes the active role of firms as accelerators of global transition processes, and based on qualitative content analysis of the literature, we found key pillars of CE (governance, relations with stakeholders and innovation) that SMEs should manage in an integrated way to increase the speed of the transition towards circularity. The result of this study is a conceptual framework that explains the development of the identified pillars in the context of the transition towards CE. This study addresses a gap in the literature concerning SMEs’ transition towards circularity, emphasizing the importance of a dynamic vision and the integrated management of a variety of key dimensions. The study also provides pragmatic advice to facilitate self-assessments by SMEs with respect to their path of transition and to maximize the effectiveness of policy-makers’ interventions to support SMEs. Finally, the study has societal implications: promoting the transition of SMEs towards CE can accelerate the global green transition due to the proximity of SMEs to the local environment and work force.

## Introduction

The health emergency associated with COVID-19 and the current situation of material price volatility, resulting from high demand and limited supply due to resource scarcity, highlight the vulnerability of the traditional model of development with regard to the protection of the environment, the economy and people’s wealth, thus emphasizing the importance of resilience in generating economic opportunities while returning environmental and social benefits. The shift from a linear to a circular economy (CE) can be a legitimate answer to these many issues.

CE is a more sustainable way of managing natural resources that incorporates a regenerative system (Bocken et al., 2016; European Commission, 2015; Ellen MacArthur Foundation, 2013; Korhonen et al., 2018a; Pearce & Turner, 1990; Webster, 2015) and is characterized by a closed loop (Geissdoerfer et al., 2017), in contrast to a linear economy consisting of the following steps: Take, Make, Use and Waste (Ormazabal et al., 2018). The main frameworks for the strategies on which CE is based are known as the 3Rs (Reuse, Repair, Recycle), the 4Rs (which also includes Refurbish), the 6Rs (also including Rethink and Remanufacture) and the 9Rs (which adds Repurpose, Recover, and Reduce) (Salvioni et al., 2022a). Therefore, the loop between production and consumption can be closed into a circle by means of the addition of ‘Reuse’ (according to which consumers use previously discarded products once again while maintaining their original functions), ‘Repair’ (which refers to instances when products are restored to good conditions for reuse), ‘Recycle’ (indicating that products’ materials are processed to produce other products), ‘Refurbish’ (which occurs when products are updated to recover their original qualities), ‘Rethink’ (such as when products are used more intensively or are transformed into services, i.e., rented instead of sold), ‘Remanufacture’ (indicating that parts of discarded products are used in new products), ‘Repurpose’ (which refers to occasions when discarded products are used as parts for new products), ‘Recover’ (suggesting that discarded products are used as sources of energy), or ‘Reduce’ (which highlights situations in which products are made with fewer resources and materials).

As Homrich et al. (2018) note, CE is a win‒win strategy that can solve the problems of resource scarcity and waste by highlighting a new perspective on the potential value of all the processes in the chain. Upstream, there is a need to manage resources more efficiently, i.e., to increase the productivity of the production and consumption processes, reduce waste, and maintain the value of products and materials to the greatest extent possible. Downstream, it is necessary to ensure that everything that continues to have a residual and intrinsic usefulness is not discarded in landfills but rather recovered and reintroduced into the economic system. These two aspects constitute the essence of CE, allowing one to close the loop constituted by the value chain of products and materials. According to the European Green Deal Strategy, CE can help combat climate change, overcoming approximately 50% of the gap that separates us from reaching a temperature target of 1.5 °C.

The term circular economy was first formally used by Pearce & Turner (1990), who emphasized a conception of our planet as a closed system where everything serves as an input for everything else. Since that definition, many other attempts have been made to define CE (Kirchherr et al., 2017; Korhonen et al., 2018b), focusing on the efficient use of resources and elimination of waste (Rizos et al., 2017), increasing the lifespans of products as much as possible (Morgan & Mitchell, 2015), the resilience of socioecological systems (Gallaud & Laperche, 2016), the net positive environmental effect (Braungart et al., 2006), and the concept of zero emissions (Pauli, 2010). Korhonen et al. (2018b) highlight the importance of considering CE from a production–consumption perspective and define CE as ‘a sustainable development initiative with the objective of reducing the societal production-consumption systems' linear material and energy throughput flows by applying materials cycles, renewable and cascade-type energy flows to the linear system’ (p. 547). The fact that different stakeholders are interested in CE implies the impossibility of establishing a single universal definition that includes all these dynamic and evolving interests.

The realization of the potential of CE, in fact, requires commitment from multiple stakeholders (such as policy-makers, industries, companies, and individuals) to facilitate a process of transition that involves business, societal values, norms and behaviours (Chizaryfard et al., 2020). In this shift towards a more sustainable world, small and medium enterprises (SMEs) play a pivotal role (Eurostat, 2020), as noted by the European Union, which aims to become the world leader in CE via the enhancement of SMEs (European Commission, 2020).

Therefore, the green transition based on CE cannot be the responsibility only of large corporations. Some of the world’s largest companies have started to embrace CE as a way of creating economic value and achieving social and environmental targets (e.g., Enel, Eni, Renault, Intesa San Paolo, H&M, Mitsubishi, Philips and many others). Research suggests that firm size is an important factor in determining the extent and quality of sustainability practices; that is, smaller firms engage in fewer environmental practices (Brammer & Pavelin, 2008). The results reported by Bassi and Dias (2020) suggest that the decision to favour CE behaviour is closely linked to scope, both in terms of employees and rates of turnover. Articles debating CE practices in large companies by employing case study methodology (Brondoni, 2020; McIntyre & Ortiz, 2016; Tagliafierro, 2020) and best practices collection by international associations (such as Ellen MacArthur Foundation) confirm the maturity of corporations with regard to CE.

The favourable attitude of large companies towards CE is also justified by the previous endowment of resources including financial, tangible and intangible assets as part of the governance approach. In fact, the search for competitive advantage in global markets has long functioned as a stimulus for encouraging efforts aimed at guaranteeing sound and sustainable governance (Salvioni et al., 2022b) (e.g., by means of special committees among various boards of directors that are devoted to sustainability issues and the establishment of effective risk management systems to reduce risks associated with the dimensions of ESG), transparent communication with stakeholders to strengthen those relations (e.g., by means of integrated reporting and structured stakeholder engagement projects), and continuous attention to innovation in terms of products, processes and business models. For these reasons, the approach of corporations to CE is not entirely new, but it remains part of a wider effort to ensure sustainability that began some time ago. In other words, large companies are already equipped with a basic stock of resources and competencies to accomplish a complete transition to CE and instead need, if anything, circular tools and frameworks pertaining to specific issues (e.g., accounting, budgeting, risk management, and the assessment of investments).

Although large companies are more organized with respect to handling the shift to the CE in a holistic manner, the whole value chain (both upstream and downstream) must embrace the values of circularity due to the need to engage with the companies of suppliers and customers, even if those companies are smaller in size. However, some characteristic elements of SMEs, such as the notion of the owner-manager and informal processes of relations and communication, facilitate the use of informal sustainability practices, causing the transition to formalized and structured policies to be difficult in this context (Perrini et al., 2007; Russo & Tencati, 2009).

In this way, the approaches taken by SMEs to social responsibility and sustainability, even if they exist, tend to remain implicit or silent (Jenkins, 2004; Matten & Moon, 2004; Ormazabal et al., 2018). As a result, SMEs suffer from internal inadequacy with regard to addressing the transition towards circularity coherently (Geng & Doberstein, 2008; Ormazabal et al., 2015) and must be provided with specific support and knowledge that can facilitate the transformation of sustainability-oriented practices into a consistent strategy of rethinking the business from a circular perspective (Gennari & Cassano, 2020).

This paper refers to transition management (TM) theory as its theoretical background (Hernández-Chea et al., 2021; Loorbach & Wijsman, 2013; Van Bakel et al., 2009) for understanding the process of transformation from a traditional or linear economy to a circular economy. Transitions are complex phenomena that feature a shift from one dynamic equilibrium to another, involving different actors and requiring the identification of key factors that can catalyse very different stakeholders (Loorbach, 2007). TM theory claims that understanding transitions allows us to anticipate the shifts that can influence theirs speed and direction.

The pandemic experience associated with COVID-19 has extraordinarily increased attention to ecological and societal issues and highlighted the need for disruptive changes at both the macro and micro levels (Dolnicar & Zare, 2020; Lozano & Barreiro-Gen, 2021; Srisathan & Naruetharadhol, 2022). The shock resulting from the COVID-19 pandemic has also resulted in a rapid increase in the growth of small businesses (Litton & Solomon, 2017). Accordingly, SMEs should be aware of their pivotal role in the shift towards a more global green economy and should manage their transition processes actively. The ability to view the path towards sustainability as a transition that must be managed at both the global and firm levels is important for firms themselves, which should be aware of their transformative role in structural global changes, as well as for regulators, policy-makers and institutions, which should adjust their efforts to support circular paths by adopting a dynamic approach that is consistent with the firms’ evolution in the context of sustainability.

This paper, in accordance with TM theory, aims to identify the key factors that SMEs should manage to increase the speed of their shifts towards CE. Focusing on firm-level key factors pertaining to the transition from a linear state to a circular one, we include a critique of TM related to firms’ ability to directly manage themselves with respect to achieving the desired outcomes (Kemp, 2007). In particular, we aim to investigate the following question: *what are the main and fundamental pillars that must be managed by SMEs to accomplish a strategic transition towards CE?* Answering this research question can allow us to identify the factors that catalyse the attention of both SMEs that are engaged in CE transitions and policy-makers who desire to support SMEs’ shifts towards circularity. This paper aims to offer a novel conceptualization of the CE transitions of SMEs by developing a conceptual framework that can help us understand the CE shift at the micro level as a complex process that must be managed in different ways according to the different steps involved in the transition (Markard et al., 2012).

Numerous publications concerning CE have emerged over the past decade. Nevertheless, less research has highlighted ways of implementing a circular approach in SMEs during the transition towards CE (Cramer, 2020).

CE has been studied at the micro level from an admittedly fragmented variety of perspectives with regard to the drivers and barriers to CE (Clark et al., 2016), circular business models (Geissdoerfer et al., 2020; de Sousa Jabbour, 2019; Suchek et al., 2022), circularity and sustainable development targets (Bag et al., 2021; Dubey et al., 2019; Tang & Liao, 2021), and circular assessment tools (Life Cycle Assessment, Material Flow Analysis, Material Flow Cost Accounting). Thus, efforts to understand and synthesize the transition of SMEs to CE are required (Zhu et al., 2022).

Other comprehensive frameworks for CE have been developed to aid its conceptual development and practical implementation, especially in terms of CE-related readiness assessments on the part of SMEs.

Awan & Sroufe (2022) propose a conceptual framework to investigate the impact of obstacles and enablers on an organization’s readiness to transition to a CE-based business model in a particular sector (reuse commodities). These authors identify some actions pertaining to production and management that can accelerate the innovation of business models with respect to sustainability. De Sousa Jabbour (2019) provides insights into the factors associated with SMEs’ readiness to shift towards CE, focusing on the need to establish better relationships and shared values between consumers and organizations. The framework introduced in that paper explains three levels of analysis that organizations can use to understand the ways in which they can enhance their readiness to embrace a circular economy: the market environment, the organization, and the managerial decision-making level. However, although that study suggests combining an awareness of the market environment and the organizational changes to be applied to reshape technical and managerial decision-making, it does not emphasize the path of circular transitions and lacks a dynamic vision, thus failing to offer any contribution to the research concerning the ways in which a firm can change its mind-set, skills and relationships during the journey from LE to CE (Thorley et al., 2022). Sharma et al. (2020) gather information concerning prospects, impediments, and readiness prerequisites for the transition from LE to CE by means of a survey of representative Indian SMEs, reporting that the analysed SMEs were concerned about the environment but unaware of CE-related practices and terminology. SMEs reported a lack of guidelines pertaining to the implementation of CE as well as a lack of monitoring tools for assessing their current and future performance. Similarly, Kazancoglu et al. (2020) highlight the need for a holistic framework to successfully implement CE throughout the entire supply chain. Firms in the value chain should be able to evaluate the performance of their circular practices. The absence of points of reference and the complexity of existing tools for measurement and monitoring indicate a misalignment of metrics in the context of sustainability practices, which are the primary source of inefficiency and disruption in supply chain interactions (Björklund et al., 2012).

Some conceptual frameworks focus on organizational issues more closely to understand firms’ readiness for CE, such as the framework proposed by Holt & Vardaman (2013), which highlights three main areas: “individual factors” (the characteristics of those being asked to change), “structural factors” (the circumstances under which the change is to occur) and the “level of analysis” (whether the individual or the organizational level). Additionally, Eikelenboom and de Jong (2021), by reference to a sample of SMEs in the Netherlands, develop and test a model offering insights into the organizational attributes that can assist firms in implementing circularity in their business strategies. Reporting the absence of comprehensive change readiness models in the context of manufacturing SMEs, Thorley et al. (2022) provide a conceptual model that can allow both practitioners and researchers to understand SMEs’ adoption of CE. Although the novelty of this model is related to its combined examination of individual readiness for change and the collective perspective of readiness, its applicability to the adoption of CE by SMEs is limited to its use as a preparatory step.

Zhu et al. (2022) develop a transformative conceptual model for SMEs in emerging markets using a multilevel perspective to understand the evolutionary paths of CE and relevant circular practices. Although these authors construct a transformative model that emphasizes the ongoing transitionary process underlying SMEs’ approaches to CE, they admit the complexity of the multilevel perspective adopted in the study.

Chen et al. (2020) compare the integrated frameworks used for circular business model studies in the context of transition flow processes proposed by BSI (The British Standards Institution), WBCSD (World Business Council for Sustainable Development), and the C2C BIZZ project (Cradle to Cradle), focusing on the positioning and roles of analytical tools in these contexts.

Thus, to our knowledge, there is a gap in the frameworks suggested by the literature due to the partial approaches to the CE transition taken by previous researchers. The distinctive contribution of our model lies in the fact that it presents a strategic and comprehensive view of the ways in which SMEs can evolve towards CE by means of their own management of key pillars of CE. Awareness of these factors and the ability to manage them can impact the manner and duration of the transformative transition towards circularity.

This paper contributes to both academic research and the practical shift of SMEs towards a strategic approach to CE. The paper advances the progress of CE research because it represents a novel approach that ranges beyond mere criticism of existing frameworks for SMEs. Moreover, the paper provides a new firm-level conceptual model of SMEs’ transition towards CE with potential practical impacts for both SMEs and policy-makers. Supporting SMEs in their shift towards CE also has a social impact accelerating the green transition.

This paper is structured as follows. We present the paper’s theoretical background and extent studies concerning CE and SMEs to highlight the scope of this contribution and its novelty with respect to previous research. Subsequently, we describe the methodology used to answer the research question. We present the results by developing a conceptual framework and discuss the findings, emphasizing theoretical, managerial and societal implications. Finally, we provide conclusions alongside both limitations of this study and suggestions for future research.

## Theoretical background and literature review

Transitions are complex phenomena that involve a shift from one dynamic equilibrium (such as a linear economy) to another (such as a circular economy). TM theory was introduced in the context of the debate concerning sustainability to address problems associated with complex challenges to societal sustainability (Van Bakel et al., 2009). In fact, transitions—shifts resulting in nonlinear changes in cultures, structures and practices—are the results of a long and complex process that involves different aspects of life, such as the economy, technology, society, and ecology (Grin et al., 2010). This complexity implies that transitions are very difficult to predict throughout their evolution; however, they can be anticipated (Rotmans et al., 2001). TM theory is based on the belief that understanding transitions allows us to anticipate the shifts that can influence their speed and direction.

Sustainability is a complex and societal transition that occurs at a global scale and offers a new perspective and new tools for management (Van Bakel et al., 2009). This co-evolutionary (rather than a revolutionary) approach to change is adopted with respect to the path-dependency principle (Avelino & Rotmans, 2011), which includes awareness of the fact that global changes are the result of small-scale actions. Transition processes are, therefore, based on the adaptive or proactive capabilities of actors at various levels, but they remain linked by the need to operate in a context that is characterized by transition pressures.

The transition approach includes some generic and global issues as drivers in different contexts to create bottom-up solutions. The two related branches of the transition literature are the Multi-Level Perspective (MLP) approach (Zhu et al., 2022), which views transitions as outcomes of alignment among actions at multiple levels (micro, meso, and macro), and the Transition Management (TM) approach, which claims that the direction of transitions can be managed deliberately and influenced by actors. This task implies identifying and aligning the key factors according to which transitions can be catalysed by very different stakeholders (Jackson et al., 2014). Transitions to a circular economy involve both approaches. International institutions engaged in the transition towards CE must address the task of defining various aspects of CE (for example, reference standards, metrics and indicators, business model frameworks) at different levels (MLP). Firms that are familiar with their role in the shift towards a more circular world and aware of the different stages that must be managed to ensure a structured and organized transition towards CE are more ready to face the transition by influencing the speed and effectiveness of the transition itself at a global level (TM). A transition can further be described as a management approach that involves changing the conditions of the traditional business model to suit another system with different requirements (Awan & Sroufe, 2022).

Therefore, it is necessary to take the perspective of TM theory as the theoretical foundation of our research because this framework can be used both to analyse and to structure or manage the ongoing processes in society, viewing sustainable development vision as a long-term goal and focusing on ways of influencing, coordinating and mainstreaming the relevant actors and their practices (Loorbach & Rotmans, 2010). TM affirms that the actors who are involved in a complex process of change at different levels (such as the attempt to transition towards more sustainable socioeconomic growth models worldwide) can develop and implement strategies that can influence this process. Attempting to steer a transition process from the outside is not effective: structures, actors, and practices must be adapted and anticipated in a manner that it must be directed from within. The TM model identifies three types of transition management: operational transition is based on practices, tactical transition focuses on structures, and strategic transition involves cultural aspects (Loorbach, 2007). The system that subsequently emerges is a multilevel network in which actors, occasionally even unconsciously, contribute to achieving shared goals by using various types of strategies and actions. That is, every business, whether large or small, can participate in implementing CE in different ways. Therefore, great attention must be devoted to entire supply chains, considering the fact that SMEs account for 99% of enterprises (Lessidrenska, 2019) and often serve as suppliers for bigger companies. While many large companies have already implemented a culture of sustainability and adopted related strategies (Vovchenko et al., 2020), SMEs must still be encouraged to transition from circular practices to circular strategies to accelerate the green transition at multiple levels (Prieto-Sandoval et al., 2018).

While these considerations can serve as a sound foundation for the use of TM, they are not free from criticism. Such critiques include the claim that many transitional developments have been unintended and initially unforeseen, thus qualifying them as spontaneous changes, and scepticism concerning the role of a guiding vision, considering the fact that many historical transitions have not been led by general visions of the future (Berkhout et al., 2004). Our conceptual framework aims to overcome most of these challenges by placing the transition of SMEs in the context of a broad global vision (started with Agenda 2030), which is realized in accordance with the top-down and bottom-up approach that is typical of the TM (Rotmans et al., 2007): the top-down aspect refers to the Agenda process (e.g., the EU Circular Economy Package), whereas circular experimental practices by companies (e.g., the energy circularity of a site for companies operating in the energy industry) are typical bottom-up aspects.

Based on this theoretical background, we discuss the ways in which the TM takes shape in practice.

Transition theories have already suggested reference frameworks that have been criticized due to their disregard of the firm-level perspective (Brendzel-Skowera, 2021; Mendoza et al., 2017). The framework developed by Rotmans and Loorbach (2009) proposes the existence of four stages in a TM cycle and emphasizes that transition experiments are initiated by microlevel actors; however, this framework has also been subjected to criticism due to its limitations resulting from its insufficient consideration of multisystem interactions (El Bilali, 2020; Hoppe et al., 2016).

Some academic contributions claim that the transition process exhibits different characteristics in different stages and may require a variety of approaches and forms of resource/knowledge support (Holzer et al., 2021), thus suggesting the use of different schemes for conceptualizing firms’ transitions towards sustainability. Brendzel-Skowera (2021) identifies five stages of transition with respect to a situation in which environmental actions are encouraged by pragmatic reasons to achieve a full implementation of CE on the basis of relations with stakeholders. For Ormazabal et al. (2015), the number of stages involved in this process is six. Many other authors address the attitudes that firms could adopt with respect to a change process (Chen et al., 2020; Girotra & Netessine, 2011; Holzer et al., 2021; Jabbour, 2010; Klewitz & Hansen, 2011; Noci & Verganti, 1999; Saidani et al., 2017), thus indicating a continuum that can be summarized in terms of resistant, reactive, proactive and innovative attitudes. Resistant attitudes can cause SMEs to ignore the pressures to improve their environmental performance, thus resulting in a nonstrategy. Reactive strategies mainly involve reactions to external stimuli caused by governments and regulators, stakeholders, and other firms. Proactive strategies imply a deliberate effort to take advantage of organizational capabilities and firm characteristics in response to the opportunities offered by the environment. A more mature approach is the use of innovation-based strategies, which involve a fundamental rethinking of all aspects of the business, from structure to management, in a holistic way. In this case, the environmental variable is the most important competitive factor, and the introduction of new radical technologies is emphasized.

Studies in the field of transition that focus on the micro level are generally limited by their adoption of a static perspective and lack an explanation of the ways in which firms progress from one stage of the transition to the next (Jabbour, 2010; Ormazabal et al., 2015) as well as an integrated vision of the business in the context of a general concept of sound governance (Loorbach, 2007), in which case CE is a key long-term factor related to success.

The literature also emphasizes the importance of long-term strategic thinking in progressing towards solutions that are not limited to mitigating environmental and societal impacts but rather allow us to restructure and rethink traditional modes of production and consumption. While the literature pertaining to CE is abundant, specific research concerning the topic of circular transition remains scarce (Bassi & Dias, 2019), with particular with regard to SMEs. The same literature emphasizes the need for the development of a framework that can offer support to companies in their paths towards CE in a comprehensive manner (Korhonen et al., 2018a; Rizos et al., 2016). Elia et al. (2017) reviews the methodologies presently in use, showing that none of these approaches in isolation is capable of monitoring the characteristics of CE. In addition, these studies require tools and skills that SMEs do not often possess and are generally too focused on particular activities in the value chain, which are mostly related to the level of circularity in the flow of materials during the production processes (circular inflow and circular outflow); accordingly, such studies do not adequately emphasize the importance of managing the circularity of the business in a systemic and strategic manner (Geng et al., 2012; Li, 2012; Saidani et al., 2017).

SMEs exhibit positive attitudes towards sustainability practices (The Globalization Council, 2009). Thus, by adopting the correct strategy, they can contribute to the task of promoting green global growth, the protection of the environment, and the solution of societal challenges (Yadow et al., 2018). Despite their potential, SMEs struggle to manage their sustainability commitment consistently because of a variety of barriers (Aghelie, 2017; Bassi & Dias, 2019; de Jesus & Mendonça, 2018; Musa & Chinniah, 2016; Oncioiu et al., 2018; Pheifer, 2017; Rizos et al., 2016; Tura et al., 2019; Yadow et al., 2018) catalogued by Kirchherr et al. (2018), including cultural, regulatory, market and technological barriers.

A sustainability transition, therefore, is based on an understanding of the process of transforming from a traditional or linear economy to a circular economy that can transform barriers into potential drivers of change.

## Research methodology

The research question guiding this study is as follows: *what are the main and fundamental pillars that must be managed by SMEs to accomplish a strategic transition towards CE?* Answering this question highlights a few fundamental concepts on which the transition from a linear to a circular economy must be based according to an integrated view of firms that maintains that CE cannot be accomplished through a piecemeal approach. The reference to transition theory emphasizes the fact that the management of these pillars by SMEs during their shifts towards circularity is key to accelerating this shift by providing adequate knowledge and resources, as the transition process exhibits different characteristics across different stages of maturity (Holzer et al., 2021). The intended result of this study is the development of a conceptual framework that contributes to the literature concerning CE and SMEs and that can also serve as a point of reference that can offer SMEs a qualitative firm-level tool to monitor their advancement towards CE and can allow policy-makers to attain a view of SMEs’ progress towards circularity and the supporting measures that are thus necessary.

To address this research question, the following objectives are proposed:

I. To identify the key drivers of CE, the attention of SMEs must be catalysed, and these drivers must be sorted into categories.
II. To analyse the approach that should be taken to the categories thus identified during the transition process in accordance with the awareness of CE on the part of SMEs.

For our purposes, we employ a qualitative research method because we consider this approach to be useful for conceptualizing research as the process of reducing uncertainty and fragmentation with respect to our knowledge of important phenomena. One way of concretizing this approach is to characterize it in terms of open-ended questions to facilitate the development of conceptual frameworks or theories (Sofaer, 1999). Denzin & Lincoln (2008) claim that qualitative research involves interpreting phenomena in terms of the meanings with which people associate them. Considering the fact that CE is an evolving concept that continues to be defined in unique ways, research concerning this issue should be flexible, taking into consideration the context in which the research occurs (Green & Thorogood, 2004). In this sense, we share the belief that qualitative research is cyclical in nature (Flick, 2009; Maxwell, 2005); that is, during the research design step, a deductive approach is employed because the concepts of existing theories are incorporated into the methodology, while an inductive attitude is prominent in the data collection step, thus reflecting the perspective of grounding analysis (Hennink et al., 2020).

**Fig. 1:**
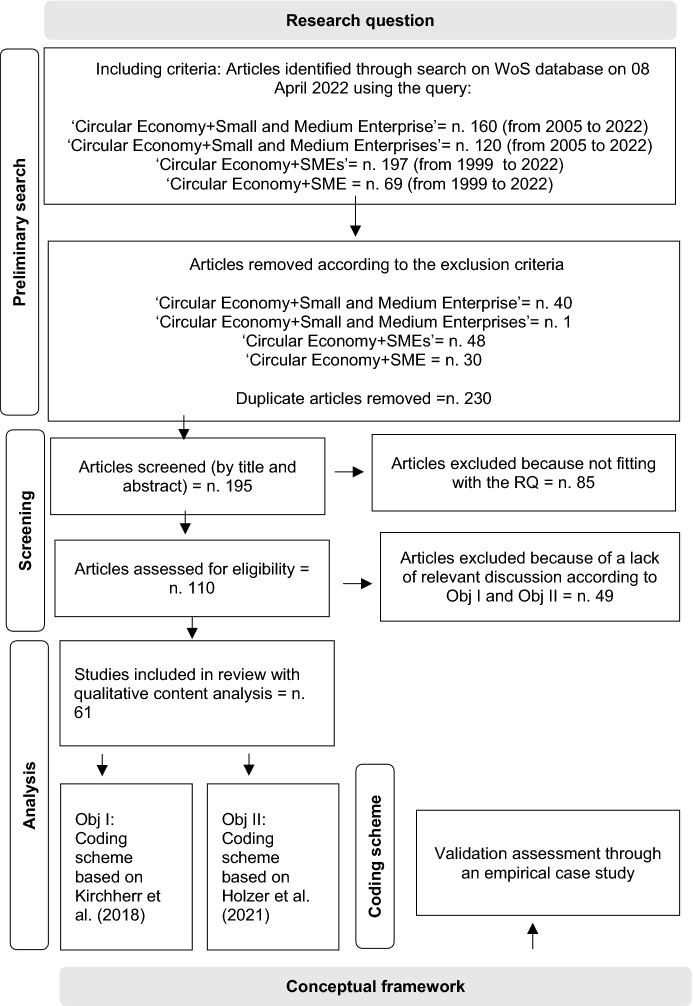
Methodology for the literature analysis

To accomplish these objectives and solve the research question at hand, a literature review was performed to synthesize existing knowledge concerning CE in SMEs from the fragmentary research. The contributions included in this review were selected as the subject of our analysis in accordance with the Preferred Reporting Items for Systematic Review and Meta-Analysis (PRISMA) approach (Moher et al., 2009) (Fig. 1).

*Step 1. Preliminary search*: We performed a preliminary search using the Web of Science (WoS) database to identify contributions related to our research. WoS was the first broad-scope international bibliographic database to emerge, and over time, it has become the most influential bibliographic data source used for journals selection and research evaluation (Li et al., 2018). Compared to Scopus, WoS provides more thorough coverage of older literature (Pranckute, 2021). We used the following query strings in all fields: ‘Circular Economy + Small and Medium Enterprise’, ‘Circular Economy + Small and Medium Enterprises’, ‘Circular Economy + SME’, and ‘Circular Economy + SMEs’. We considered only published contributions (articles, review articles, and book chapters) and excluded proceedings papers. We retained publications from relevant WoS categories such as environmental sciences, green sustainable science, environmental studies, environmental engineering, business, management, economics, and development studies. We excluded contributions that were excessively focused on very specific sectors (such as forestry, biotechnology, agronomy, biochemical, chemistry, and civil engineering) and obtained 546 results. Subsequently, we removed any duplicates, resulting in a total of 196 contributions to be screened.

*Step 2. Screening*: These publications were analysed directly by reading their abstracts to assess their eligibility for the final step in the review, which focused on the design of the conceptual framework using a qualitative content analysis approach. We discarded documents that did not pertain to our research question and research objectives (that is, articles consisting of technical information or those that focused on a macrolevel of analysis in terms of countries or regions). A total of 86 contributions were excluded because they were inconsistent with the research question. A total of 110 contributions were submitted to such abstract analysis. Regarding Objective I, we followed the suggestions of Scipioni et al. (2021), who selects publications regarding CE barriers and/or enablers because in a sustainable-oriented transition of SMEs, many factors that are defined as barriers can also act as drivers for the activation of sustainable processes. Regarding Objective II, we employed the approach used by Holzer et al. (2021), who contribute to the systematization of previous studies concerning the path of transitioning towards CE on the part of SMEs by identifying meaningful clusters depending on the perceived importance and performance in various topical areas of CE. Thus, we retained publications that pertained to the approaches used by SMEs to transition towards CE.

This screening phase allowed us to exclude contributions that did not feature relevant discussion of issues related to our research objectives. Sixty-one contributions were advanced to the subsequent step of the process.

*Step 3. Analysis*: Sixty-one articles were included in the final review, which employed the method of content analysis. We considered content analysis to be as a useful method for enhancing our understanding of a phenomenon via the analysis of textual data (Elo & Kyngäs, 2008). Using this technique, we were able to produce a conceptual description of the state of the art of the research concerning the topic at hand (Munn et al., 2018; Sargeant et al., 2006), thus enabling us to meet the need to overcome the fragmentation of studies concerning CE in the context of SMEs. Chen suggests the importance of using a focused and integrated approach (Chen et al., 2020) with respect to the issue of CE transition, and as Siegel et al. (2019) note, the development of a simplified and generic framework to combine various aspects of sustainability can help understand the matter at hand and overcoming the existing shortcomings in the literature. We conducted research using a directed approach to qualitative content analysis (Hsieh & Shannon, 2005), using previous research findings as a guide for our coding. To overcome the limitations of directed content analysis, which mainly pertain to the fact that researchers might be more likely to discover evidence that is supportive of a theory rather than evidence that contradicts it, we desired to ensure the reliability of our study. Reliability requires the same results to be obtained if the study is replicated (Morse & Richards, 2002). Accordingly, we developed a deductive coding scheme from conceptual propositions (Catanzaro, 1988) to make the process leading from the data to the results transparent, thereby minimizing the cognitive changes that occurred during the analysis. This coding scheme allowed us to sort general constructs into intellectual “bins” (Miles & Huberman, 1994), thus linking data to propositions (Yin, 2003).

*Step 3.1 Coding scheme for objective I*: To code qualitative data inductively, we defined a content guide based on the findings of Kirchherr et al. (2018), who, with the aim of balancing comprehensiveness with parsimony, organizes previous research concerning the barriers to/potential enablers of CE in the context of SMEs by distinguishing among cultural, regulatory, market and technology barriers/enablers.

Cultural barriers are mainly related to the notion that companies have not yet mainstreamed the full extent of CE. Culture refers to a set of values, beliefs and behaviours that are shared within a firm influence all aspects of the governance of the business. Market barriers refer to the inability of markets to perceive the future opportunities for CE, focusing instead only on current economic considerations (e.g., the fact that fossil fuel-based plastics are much less expensive than bio-based plastics). We traced these barriers back to the firm’s general relations with stakeholders. Responsible production and consumption typical of CE is promoted by the ability to create and maintain virtuous interactions with upstream and downstream actors throughout the value chain, thus enabling the creation of a market in which all players feel involved concretely in the achievement of collective objectives according to a win‒win logic. Technological issues refer to the ability to change the firm’s usual mode of operation. Innovation serves as a foundation for and accelerator of sustainable development as a result of disruptive technologies that enable changes in terms of time and cost that would have been inconceivable decades ago. Regulatory barriers refer to policy-makers’ actions with respect to the implementation of obstructing laws and regulations. These barriers are not viewed as critical in the research by Kirchherr et al. (2018), even if governments could do more to accelerate firms’ transitions towards CE. We do not consider these barriers in our analysis because they are not subject to the direct control of firms and can differ depending on time, country, and industry.

**Fig. 2:**
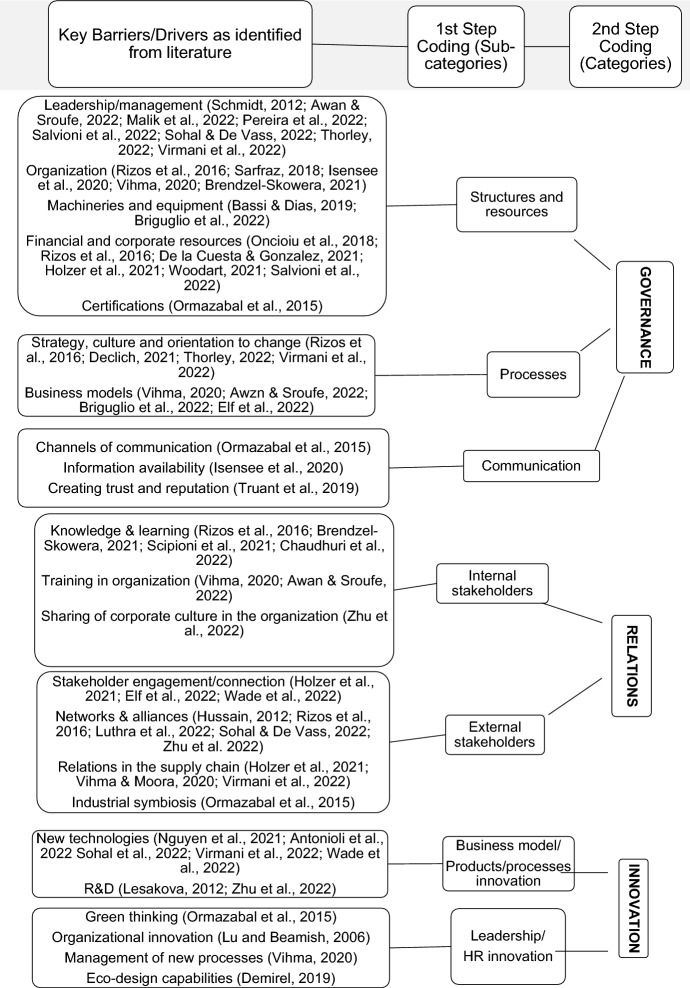
CE key factors—coding frame

Analysis of the literature allowed us to collect a series of factors that can as barriers to or potential enablers of the application of CE by SMEs. We excluded factors that were not subject to the direct control of firms (for example, governmental actions or competitors’ behaviour), and we created codes that accounted for all remaining factors cited by the authors and standardized synonymous factors (for example, organization, staff, human resources, organizational structure). The resulting coding frame is depicted in Fig. 2 and pertains to the first objective of this research.

**Fig. 3:**
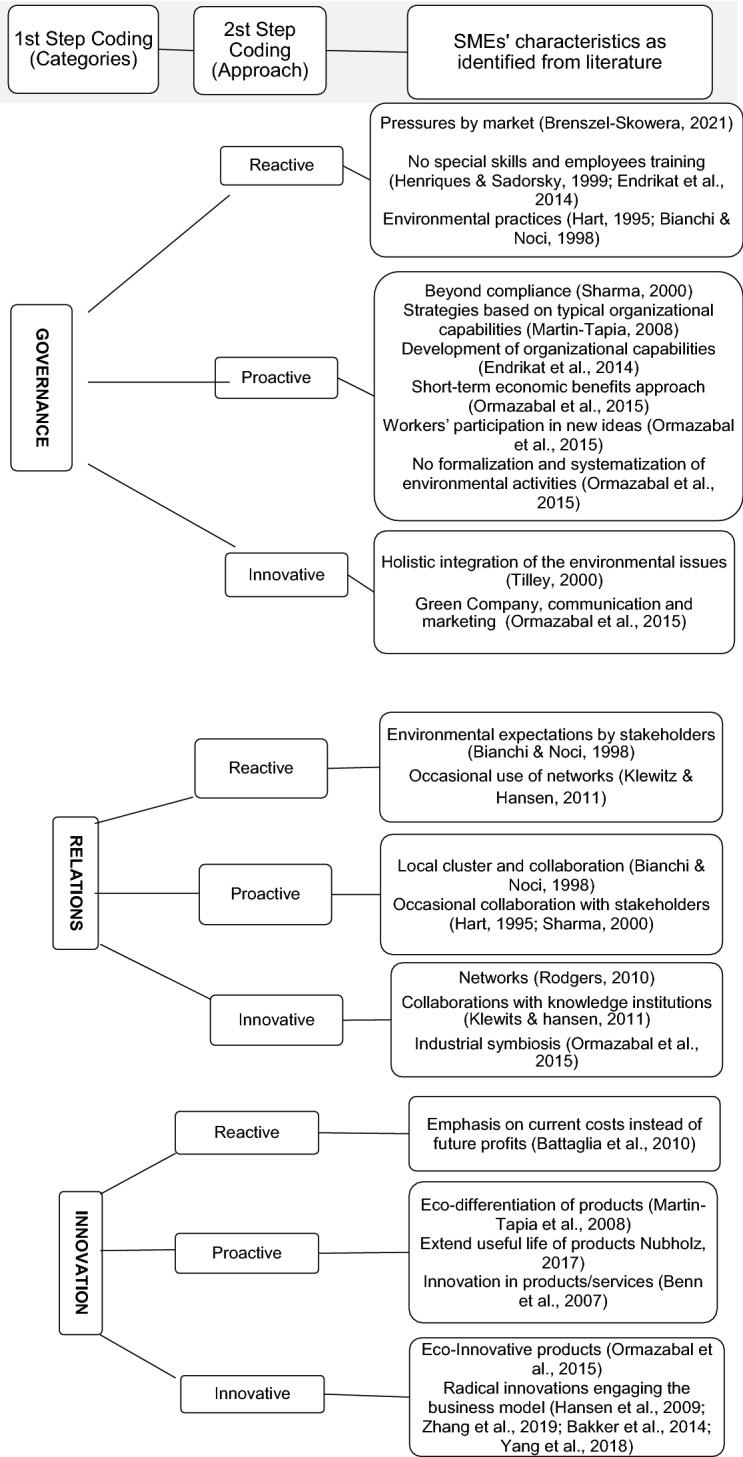
CE approaches—coding frame

*Step 3.2 Coding scheme for objective II*: The following step in the research, which is related to the second objective of the research, pertained to the search for information regarding the different attitudes displayed by SMEs regarding CE in accordance with previous studies concerning the process of transition towards CE, as codified in Fig. 3. Holzer et al. (2021) systematize previous research concerning SMEs’ path of transition towards CE, identifying the following clusters. ‘Laggards’ refer to companies that do not adapt to a CE unless they are forced by legislative measures to do so. We associate this reluctant behaviour with the resistant approach (Tilley, 2000), which we do not include in our coding scheme because it does not recognize the natural importance of change, which characterizes firms as vital systems (Golinelli, 2012). The cluster ‘late majority’ includes companies that are interested in CE issues but that are unable to change their business model due to certain gaps, such as independence from resource supply or resource efficiency. We interpret these firms as reactive SMEs whose engagement in sustainability practices is influenced by external pressures, regulations from governments and authorities and stakeholders (such as consumers, green movements, or firms that operate in other sectors but that are considered to exhibit best practice that should be emulated). ‘Fast followers’ are aware of the benefits of CE, but they may require support to realize their goals and improve their circular performance. Holzer et al. (2021) note that policy-makers and economic development agencies may identify this cluster as the main target group for CE-supporting activities. We associate this cluster with proactive firms. The cluster of CE ‘forerunners’ includes companies that are very aware of CE issues and see no benefit in receiving support from policy-makers and economic development agencies. We consider SMEs that belong to this cluster to be the most innovative and to have concluded the process of transitioning to CE, i.e., as being completely circular.

*Step 4. Validation of results via empirical testing*: Finally, this framework is defined as the result of our research analysis, and we aim to validate this framework by conducting empirical research concerning a small enterprise (11 employees) operating in the bioenergy industry in the region of Lombardy (Italy).

The bioeconomy has received attention from policy-makers who, motivated by the orientation of the EU, encourage investments to reduce the use of fossil fuels with the aim of mitigating climate change and reducing dependence on scarce natural resources. The bioeconomy can have a positive impact on the transition from a linear economy to a circular economy as long as the CE includes the concept of a circular bioeconomy (European Commission, 2018). The targeted firm that we used to test our framework produces biogas, which is a technology enabling the generation of bioenergy by means of a biologically mediated process known as anaerobic digestion, according to which different microorganisms follow diverse metabolic pathways to decompose organic matter. Electricity generation from biogas in Europe increased from 3652 GWh to 88,986 GWh between 1990 and 2018 (data from International Energy Agency website, 2021). Germany is the largest producer of biogas in the EU, featuring 66% of all biogas plants, followed by Italy (10%) and France (5%) (Guidehouse Netherlands, 2020). Lombardy is the first region in Italy to invest in biogas technology with an installed power of more than 250 MV (Guidehouse Netherlands, 2020).

The case under analysis is an agricultural enterprise that has been operating since 2000 and that entered the biogas industry in 2012, which produces an average of 5 GWh energy per year. To produce biogas, this firm uses a wide variety of feedstock mostly produced on farms (such as crop residue and animal manure); this approach allows the loop to be closed in accordance with the principles of CE.

**Fig. 4:**
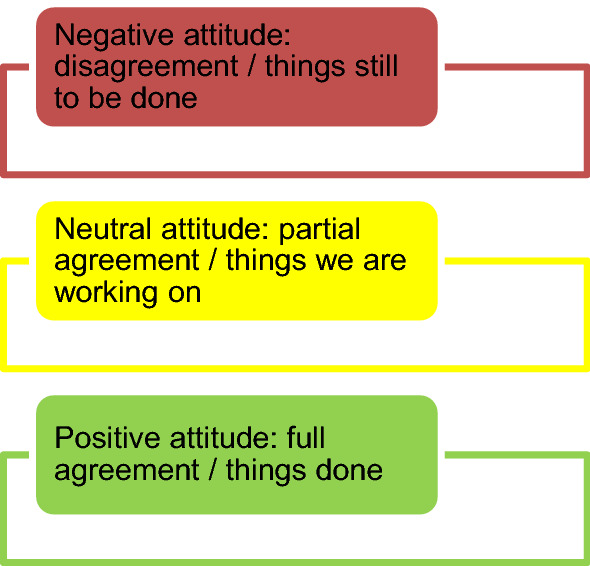
Likert scale used in the interview

We aimed to explain the conceptual framework to the entrepreneur during a face-to-face interview using an open interview method (Robinson et al., 2021). This method is based on the interviewer’s identification of key topics discussed and explored by the respondent without providing any direct questions for the respondent to answer. The role of the interviewer in these interviews is essentially to listen. Using a Likert scale, we asked the entrepreneur to indicate with different colours the firm’s attitude regarding the various issues included in the framework quadrants and to provide reasons for his answers. The possible answers ranged from a negative attitude to a positive attitude, which was intended in the sense of ‘agree with/things done’. We decide to use colours because they are more immediate, but a quantitative analysis using value scales is also possible (Fig. 4).

## Results and conceptual framework

The framework depicted in Fig. 2 emphasizes three main codes for interpretation: (a) governance, including structures, processes and information flows (which enable the transformation of resources into value for stakeholders); (b) relations, including both internal and external stakeholders (which ensure that the circular loop is closed); and (c) innovation in terms of product and related business models as well as in strategic thinking (which serves as the foundation for the transition from the paradigm based on ‘take, make and dispose’ that is typical of a linear economy to a new paradigm associated with CE). Simultaneously, the different behaviours SMEs exhibit towards these key drivers of CE (Fig. 3) highlight the various levels of maturity they exhibit with respect to circular principles.

We systematized the results of the content analysis of the selected articles in the form of a conceptual framework (Table 1) that pertains both to the fundamental factors based on which SMEs should catalyse their efforts to ensure a long-term transition towards CE and to the different attitudes that SMEs exhibit regarding these factors depending on the approach towards circularity that they adopt. We combine the three key pillars of CE that we identified (governance, relations, and innovation) with categorized transitional stages that highlight the various approaches towards circularity taken by SMEs (reactive, proactive, and innovative). According to TM theory, SMEs’ attitudes with regard to managing the key factors we identified can increase or increase the pace of the transition towards CE, ensuring the complete acquisition of the principles of circularity in terms of doing business when all three pillars are managed in an integrated and conscious way as tools for a formalized transition.

**Table 1:**
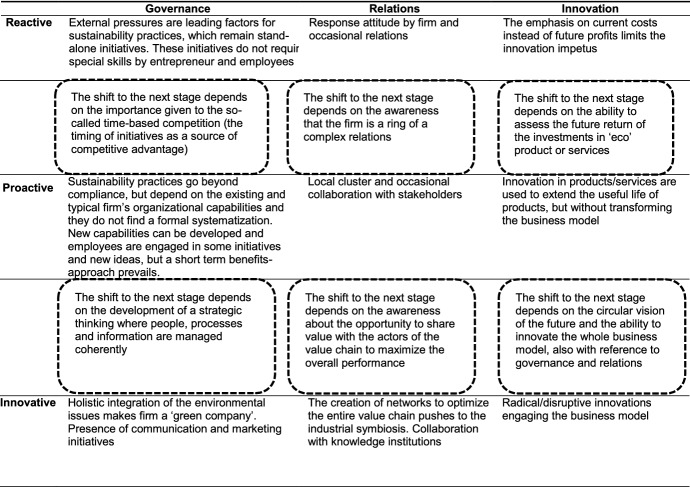
Theoretical framework

Our analysis supports the conclusions of previous studies concerning circular transition, which have, albeit in a fragmented way, already identified some key elements associated with a successful transition and highlighted the fact that-the transition to CE takes place at different levels of awareness, commitment and behaviour.

The business administration literature claims that governance presides over the orientation of all decisions pertaining to sustainable development and the related actions associated with implementation (de Jesus & Mendonça, 2018; Gennari & Salvioni, 2019; Kirchherr et al., 2018; Pheifer, 2017; Rizos et al., 2016; Salvioni & Gennari, 2017) and is thus the source of a holistic shift towards circularity. Such research provides evidence concerning the positive relationships between the sustainable development of firms and the quality of their governance, stakeholders’ pressures, and ESG corporate reporting (Almagtome et al., 2020). Our research also identified three integrated dimensions of governance: firm structure, which focuses on the firm’s leadership/management and human resources/organization (Yadow et al., 2018); corporate assets and financial resources (Ormazabal et al., 2018); intangible assets as brand image (Ormazabal et al., 2018); processes, including reference to strategic goals and business models that transform current costs and investments into both current and future value for stakeholders (Tura et al., 2019); and information flow, which also pertains to the maintenance/creation of relations with current/potential stakeholders based on trust and reputation (Truant et al., 2019). The governance of SMEs depends on their owners’ attitudes and values and can benefit from strong leadership (Kumar et al., 2014; Thorley et al., 2022). The smaller number of employees associated with SMEs makes management highly visible in this context (Ghobadian & Gallear, 1996) and encourages personal relations and the exchange of views, thus creating a potentially innovative environment (Yusof & Aspinwall, 2000). The lifestyles of SME owners affect sustainability activities in such firms (Font et al., 2016), as personal commitment to sustainability on the part of SME managers is considered to be a key driver of long-term performance (Koe et al., 2015). However, informal relations often lead to the use of informal procedures and an absence of standardization (Antony et al., 2008) with a focus on solving existing problems instead of seeking strategic opportunities (Antony et al., 2016). Hence, a shift to a structured CE for firms that have traditionally been founded in the context of a linear economy requires progress through the steps involved in this process to be inspired by engaged owners and managers (Ormazabal et al., 2018; Siegel et al., 2019).

Stakeholders’ expectations are highly relevant to the formulation of a successful strategy (Hienerth et al., 2011; Hörisch et al., 2014). A high level of engagement is recommended as a strategic approach to the effective establishment and implementation of a circular business model (Salvioni & Almici, 2020). Due to the increased awareness and knowledge of stakeholders concerning sustainability issues (Jakhar et al., 2019), firms must approach their business by considering the entire life cycle of a product, including sourcing, manufacturing, use, disposal and recovery of the product’s value after the end of this process. This situation highlights the need to integrate the capabilities of the organizational value chain with stimulating stakeholder issues from a holistic perspective (Witjes & Lozano, 2016).

Our analysis also emphasized the importance of taking both internal and external stakeholders into account. Internal relationships are based on a shared culture of sustainability, eco-knowledge, and continuous learning resulting from training. The creation of external relations and networks allows participants to join forces to increase the availability of financial/knowledge/organizational resources (Rizos et al., 2016). The phenomenon of industrial symbiosis, which refers to situations in which organizations belonging to different industries engage in mutual transactions to transform waste into resources, is considered to be a core strategy for promoting the transition towards the (Domenech et al., 2019). SMEs are highly connected within the contexts in which they are located, creating a thick network of relations with the local community. Therefore, although they often operate in the context of local SME networks, they can also be part of larger networks of suppliers (Battaglia et al., 2010). Such networks can be defined in terms of a relational environment based on systematic relations among local actors, which, however, can cause issues that are difficult to manage (for example, agreements concerning the overall budget of the network, ways of managing decision-making processes, and methods of managing overall risk) (Ferrucci & Varaldo, 1996).

Attention to innovation is increasing with respect to considering changes that affect the economic, social and environmental dimensions of companies (Brondoni, 2020; European Investment Bank, 2020; Svensson & Funck, 2019). Suchek et al. (2022) propose a framework for innovation with respect to CE, emphasizing the fact that, in general terms, such innovation depends on the establishment of strategic alliances and the adoption of a multilevel approach that includes all interested parties in terms of business model innovation and an eco-innovation approach.

Our research also identified two directions for such innovation: one such direction pertained to products and processes (included in the business model design), while the other direction was related to circular management thinking and organizational eco-capabilities. Investments in innovation with respect to business models with the aim of finding alternative solutions to issues associated with linear production must also be approached in the context of a culture focused on long-term development as well as by reference to adequate performance indicators to enable the assessment of the economic and socioenvironmental impact of such investments, awareness of financing sources (Siegel et al., 2019), and innovative methods for training and upgrading workers’ skills (Kirchherr et al., 2018; Ormazabal et al., 2018; Rizos et al., 2016; Tura et al., 2019; Yadow et al., 2018). Innovations in terms of products should be accompanied by innovations in leadership/management that offer a new vision of the future. The environmental management capabilities of SMEs allow them to respond proactively to the opportunities offered by environmental practices (Shields & Shelleman, (2015), but SMEs often suffer from a lack of financial resources (Antony et al., 2016). Consequently, SMEs tend mostly to focus on incremental innovation rather than radical innovation (Bos-Brouwers, 2009), which increases the time required for the transition towards a circular business model.

The transition towards CE engages the key pillars differently in accordance with the approaches taken by SMEs that we identified.

In general, reactive behaviour leads to the recognition of some evidence (such as environmental certifications), which contributes to the formalization of environmental management processes. At this stage, organizations do not have special expertise or skills (Endrikat et al., 2014); entrepreneurs/management are rarely involved, and no company-wide employee training or education is available because innovation is limited and considered to constitute a cost that is characterized by uncertain benefits in the future (Battaglia et al., 2010). Relations with stakeholders are restricted to a response-based attitude that allows SMEs to meet increasing environmental expectations and can facilitate their ability to overcome resource constraints (for example, by their inclusion in various networks) (Klewitz & Hansen, 2011; Liu & Yang, 2019). A firm’s way of conducting business in this context remains founded on the principles of the linear economy, although special and intentional attention is given to environmental issues with the aim of satisfying external stakeholders’ expectations and improving the firm’s competitive position.

The transition from a reactive approach to a proactive attitude regarding CE depends on the firm’s awareness of time-based competition (Brondoni, 2008)—considering the timing of initiatives to constitute a primary source of competitive advantage (Noci & Verganti, 1999)—as well as on the unique characteristics of SMEs. This awareness is reflected in the fact that SMEs in this context make more positive and deliberate efforts to reduce environmental impacts to obtain economic benefits and to satisfy consumers’ expectations without waiting for external pressure in the future. This attempt is made by recourse to interventions with respect to the product or its parts (Nußholz, 2017) as well as a review of related production processes (Martin-Tapia et al., 2008). This approach implies that such SMEs make serious efforts to increase resource productivity and material substitutions, but it allows SMEs to focus on differentiated (ecological) products and avoid competing with bigger firms. Collaboration by means of agreements and local networks is often required to compensate for the limited resources that are available to SMEs (Bianchi & Noci, 1998). This phase is also characterized by a more incisive commitment on the part of entrepreneurs/management and the spread of circular concepts among the firm’s employees to encourage a more general sense of awareness of the companies’ activities with respect to the environment. Nevertheless, these activities tend to be an ad hoc component of a sustainable approach rather than a formalized and systematized means of implementing a circular vision (Ormazabal et al., 2015).

The shift from a proactive approach to the innovative step requires the ability to envision a different future on the part of entrepreneurs/management, i.e., the establishment of this vision as the point of reference for every activity carried out by the firm (Tilley, 2000). The entirety of the firm’s business should be rethought in accordance with circular principles, for example, by developing new eco-innovative products or changing the concept of the product itself by offering a service instead of a physical good. This change of perspective is based on a high degree of commitment and involvement on the part of both entrepreneurs/management and the organization, and it requires new performance assessment tools, such as a method for assessing the life cycle of the product and innovation in terms of governance. Innovation-based solutions involve the introduction of new technologies that can improve the current competitive position of the firm radically or create new markets, such as by remanufacturing, dematerializing, or offering product rental instead of sales (Noci & Verganti, 1999; Zhang et al., 2019). This push towards radical innovation requires both human and financial resources as well as the sharing of product and process information (Rodgers, 2010) in which SMEs can engage due to their stable relations/networks with stakeholders who are part of the same value chain (i.e., symbiosis with other manufacturers and suppliers). The consumer also plays a central role in this circular process and stands as a full-fledged link in the value chain. The need for special skills at this step can be satisfied by collaboration with knowledge-focused institutions such as universities (Noci & Verganti, 1999). SMEs that have attained this level of maturity regarding CE become leading companies in the industry in their own right due to focused marketing and communication strategies (for example sustainability reporting) (Ormazabal et al., 2015).

This conceptual framework was proposed to an entrepreneur as described in the “Research methodology” section of this paper. The entrepreneur’s judgement of this framework is shown in Table 2.

**Table 2:**
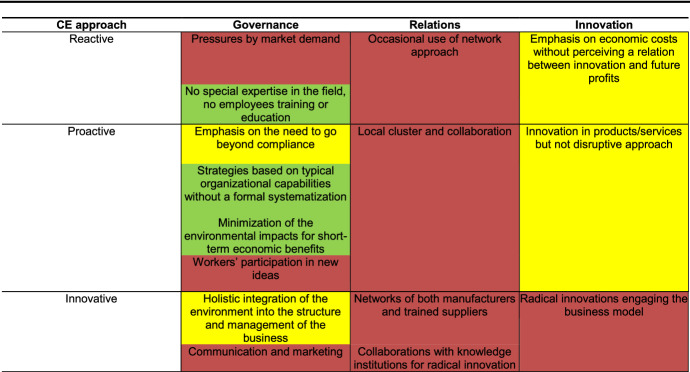
Example case

The different colours used in Table 2 correspond to different levels of entrepreneur maturity in this context. The first observation from this table is that different levels of maturity characterize different pillars; that is, the speed of transition is not homogenous within the same firm and must be managed in accordance with this fact. Nevertheless, the firm under analysis can be identified as being halfway between the reactive and proactive steps, i.e., it is committed to the circular transition but anticipates only a few challenges of and lacks an integrated approach. The entrepreneur of this firm admitted that he did not have complete knowledge of CE, and he initiated the biogas project due to its convenience (in terms of government incentives) but lacked prior knowledge of the matter as well as engagement by employees. He passed from this initial reactive step due to the pressure of an external driver (i.e., public incentives) to reach the proactive stage as a result of gaining more awareness of the benefits that the project would produce in the future, and he was aware of the importance that a radical innovation of the firm’s entire business model (biogas plant and farm) could have for the market growth of the firm.

The most critical aspect of this process is the ‘relations’ pillar, which prevents the firm from taking advantage of all the opportunities associated with biogas. The entrepreneur reported many difficulties with respect to creating relations with stakeholders. His neighbours were not yet convinced that biogas produces clean energy and were afraid of (nonexistent) air pollution. His relations with suppliers of waste products that could support a biogas plant (such as cocoa skins or pomace olive) were based on opportunistic negotiations. The absence of collaborative networks made negotiations more expensive for the entrepreneur and less effective for relevant suppliers, thus mitigating the potential benefits of this approach for the whole supply chain.

## Discussion

These results address our research question concerning the ways in which SMEs can manage their transition towards CE in accordance with an integrated and strategic approach. We uncovered three pillars (governance, relations, and innovation) that have already received attention in the business administration literature. Previous studies focused on CE in the context SMEs, however, have failed to employ an integrated approach or to emphasize the TM approach that characterizes the transformation from a linear economy to a circular economy (Chen et al., 2020; de Sousa Jabbour, 2019; Thorley et al., 2022; Zhu et al., 2022). Accordingly, we developed a conceptual framework that highlights the facts that these three founding pillars act jointly and that their management evolves throughout the transition towards CE.

Our conceptual framework demonstrates that CE cannot be a piecemeal approach to the business at hand but should rather be managed in accordance with a holistic attitude that includes different aspects that must be considered simultaneously. As a result of the literature analysis, we entered the field of CE transition while remaining open to realize new meanings, and by means of theoretical coding, we progressively became aware that certain groupings were possible and that certain patterns could be discovered, ultimately focusing on three core issues around which all the other factors could be integrated (Glaser, 1978). The three pillars that we identified should be understood as broad concepts: governance refers to the managerial/organizational structure and resources but also to a way of guiding the business and the ability to be accountable with respect to stakeholders; relations pertains to the management of knowledge, culture and information with respect to both internal and external stakeholders; and innovation regards the ability to incorporate new technologies into products and processes but also focuses on changes in terms of business approach and business thinking. The results of our analysis, in accordance with the first objective of this research, suggest that SMEs can transition from a linear to a circular approach when they are aware of the existence of different key dimensions of their business, which should be managed jointly to facilitate an effective transition.

Furthermore, the resulting framework goes beyond the critiques of immobility in the literature by emphasizing the ‘things to do’ that are necessary to progress from one stage of the transition to the next by acquiring additional awareness and an improved understanding of the real extent of CE. In accordance with the second objective of this research, our results emphasize the fact that the transition from passive or reactive behaviours to more a circular, active approach depends basically on the importance that SMEs assign to the strategic approach to the business, their recognition of the inherent value of relations, and their visions of the future. Governance, which is subject to the short-term pressures that are typical of a reactive business, can evolve through the development of circular skills and projects via a strategic approach, which is characterized by the ability to govern the future as a result of the integration of decision-making processes and activities based on clear, sustainable directions that the firm can follow. Awareness of the fact that relations can create additional value or that they are essential to the creation of value causes firms to shift from occasional relations towards networks where such value can be cocreated, that is, towards situations in which all the stakeholders belonging to the network contribute to and benefit from these relations. Innovation requires strategic thinking to develop from the stage characterized by the search for the best solutions for a reduction in current costs to the stage focused on the best solutions for the long-term sustainable development of the firm, which also requires the firm’s traditional way of conducting business to be rethought.

The case used as an example supports our conceptual framework. SMEs’ transition towards CE represents a complex and gradual path composed of different degrees of awareness and different attitudes regarding the founding pillars. Long-term circular strategies, which balance the need to offer more sustainable products with the requirement of containing costs, are the result of the intersystemic and dynamic interactions among responsible governance, the sharing of critical resources within the value chain and with internal stakeholders to create mutually beneficial relations, and the ability to take advantage of new technologies and innovations in terms of both business and the firm’s thinking.

Ecological transition at the global level is possible by developing awareness of a just transition at the local level that can be tailored to local characteristics. This fact implies that SMEs, which are the engine of growth in the EU, should be guided and accompanied in their paths towards more sustainable business models. The case used as an example emphasizes two general aspects of SMEs that are critical to this process:

- The same firm can be positioned differently with regard to the various key pillars. That is, a firm’s transition to circularity is gradual and depends on the firm’s ability and resources to govern these pillars.
- The existence of different levels of maturity (and management) with regard to the pillars of CE prevents the value chain from taking full advantage of CE and acts as a barrier to value cocreation. SMEs are not always aware of the importance of the transition towards circularity, which requires the various key aspects on which such a successful transition is based to be considered jointly. Simultaneously, stakeholders are not always conscious of their role in the value chain or of the possibility of obtaining benefits from such engagement.
- Meso- and macrolevel politics are limited in terms of their beneficial effects if they prioritize the founding pillars of CE separately, i.e., without knowledge of the different ‘speeds of transition’ exhibited by different SMEs (whether they are considered as single units or as industries). Policy-makers must, therefore, act on the various pillars supporting SMEs by implementing well-rounded interventions.

### Implications

The results of our research have the following implications. First, the key pillars emphasized in the literature as being fundamental to SMEs’ transition to CE (governance, relations and innovation) are difficult-to-implement concepts because they are rather broad with respect to the ways in which the approaches taken by the firms can differ: the grounded theory approach that we used (Heath & Cowley, 2004) allowed us to define multiple facets of these pillars, thus producing a broad definition of those pillars and contributing to theoretical research in the field of business administration. Our study addresses the need to provide a reference framework for the circular transition of SMEs, thus filling a gap reported by many authors (Jabbour, 2010; Loorbach, 2007; Ormazabal et al., 2015). SMEs can benefit from having a nonfragmented view of the transition towards CE in the context of developing a more strategic approach and a more integrated vision of sustainability via this theoretical support, especially considering the fact that, in contrast to large corporations that are already familiar with sustainability strategies as key factors in competitive success, SMEs must still be educated about these issues.

Second, the managerial implications of our research are based on the belief, supported by our results, that the path towards an integrated circular vision is to be viewed as a process of transition that is focused on a few key aspects and characterized by progressive degrees of awareness and commitment until reaching the stage of maturity, which is defined as ‘a state of achieving full development or a state of readiness of the enterprise to take specific actions’ (Munn et al., 2018).

The suggested framework is intended to serve as an easy-to-use firm-level tool to allow SMEs founded in the context of a linear economy to self-position themselves to qualitatively assess their approach to circularity and become aware of the changes that are necessary to achieve a more mature level of circular thinking in accordance with an integrated vision. The disposition of a firm’s attitude regarding the key pillars included in the suggested framework emphasizes the challenges that must still be overcome in the transition towards CE, thus making the entrepreneur aware of the current limitations and areas of strengths of the business to enable better planning for the future (which is often lacking in SMEs). SMEs should be equipped with managerial tools suitable for their complexity, and the framework that we suggest, which is based on a qualitative circular assessment, points in this direction. The use of quantitative assessment tools for circularity should be preceded by an awareness of the ways of improving the circularity of the business in all its various aspects.

Third, TM theory recommends catalysing the efforts of the different actors involved in transitions with respect to the same factors of change. According to this recommendation, this framework could also be used at the industry level (as an aggregated analysis of SMEs’ self-positioning schemes) to assess sectors’ maturity with regard to these three key dimensions. The availability of a synthetic view of the ‘state of the art’ concerning the ways in which SMEs within a certain sector approach CE in terms of the aspects that are being handled or overlooked can help policy-makers implement more focused interventions. This catalysing role could also be played by trade associations, which may have greater proximity to specific territories and local realities. In other words, an initial assessment of the approach that the SMEs with a territory adopt towards CE can activate different ways of facilitating the development of circular networks and value chains (for example, by creating platforms for meeting customers and suppliers of secondary raw materials).

Increasing the commitment of SMEs to CE offers benefits for the global green transition due to the proximity of SMEs to the local environment and work force, thus broadening the societal implications of our research. SMEs can contribute to fighting climate change. Furthermore, shifting jobs towards CE requires the translation of global targets into local strategies involving industries, supply chains, and territories and requiring collaboration across different enterprises and workers (Circle Economy, 2021).

## Conclusions, limitations and directions for future research

SMEs contribute significantly to European GDP and play a pivotal role in the ecological transition (European Commission, 2020). Scholars and both international and national institutions are working to support SMEs in this project by developing relevant knowledge and providing funds for this purpose. For their part, SMEs exhibit different degrees of receptiveness towards CE.

Many SMEs are on the path towards the implementation of CE, and researchers have developed frameworks concerning its conceptual development and practical implementation, especially in terms of CE-related readiness assessments. Nevertheless, these figures lack a comprehensive view of the active role that SMEs can play in circular transitions with regard to the management of some key pillars of CE. This study, based on TM theory and by reference to a qualitative content analysis of the literature concerning CE in the context of SMEs, highlighted key strategic pillars for the transition of SMEs towards CE in the long term. We realized that extant studies main focus, without an integrated approach, on three main issues, that is, governance, relations with stakeholders, and innovation. By adopting an integrated view of the dimensions with respect to which firms are able to manage an effective transition to CE, this work differs from previous contributions to the literature and offers a novel approach. In addition, existing studies confirm that SMEs exhibit different behaviours towards the key pillars thus identified depending on the different attitudes they adopt towards the relevant context (reactive, proactive, or innovative). The shift to full maturity in circular thinking requires an active effort on the part of firms to adopt a holistic and integrated circular approach.

In this context, our study does not aim to provide specific circular tools but rather to raise awareness of the need to change the global approach to business to enable SMEs to prepare for the inevitable changes that they must face in the near future and thus guarantee a new model of economic development.

The contributions of this study are twofold. As a key contribution to theory, this study addresses the gap in the literature concerning the application of a circular approach in SMEs and emphasizes the importance of a dynamic vision and integrated management of various key dimensions. We conceptualize the transition associated with CE from a firm-level perspective by proposing a theoretical framework that highlights the different attitudes that SMEs adopt with respect to the founding pillars of CE depending on their knowledge and awareness of circular business.

As a key contribution to practice, we suggest the use of this theoretical framework by both firms and policy-makers. In fact, SMEs can exploit the long-term benefits of CE when they attain a greater level of awareness concerning their approach to the principles of a circular economy and make an active effort to change the firm’s vision. In other words, SMEs must manage the transition and not merely undergo it. SMEs, despite their limitations, have great potential to participate in global change and must be aware of the key levers of CE that can allow them to exploit the opportunities emerging during the postpandemic period.

Awareness of the fact that a global societal transition towards sustainability is a complex process of coevolution that involves the participation of actors at different levels also highlights a debate concerning the effectiveness of the financial, technological or knowledge interventions of governments and institutions with respect to supporting the adoption of CE by SMEs. All these interventions bear fruit over the long term when they are planned in a way that encourages SMEs to voluntarily embrace the relevant global, societal and cultural changes. Finally, all stakeholders should be made aware of their contributions to the transition of modern economies towards more sustainable ways of producing, consuming and financing.

This study has the following limitations. We suggest a theoretical framework that is based on the extant academic literature, founded on qualitative content analysis and tested by reference to a single example case. Using qualitative content analysis allows the researcher to introduce relatively elementary codes, introducing simplifications that can blur a complex picture (Kracauer, 1952). Furthermore, despite the attempt to guarantee the validity and reliability of the study, the risk that different researchers could draw dissimilar conclusions from the data may remain (Bengtsson, 2016). The example case supported the proposition that SMEs display a limited understanding of the integrated approach to CE. However, a single case study is insufficiently representative of the dynamics associated with different industries and different firm dimensions. For these reasons, additional qualitative or quantitative research is encouraged to provide evidence concerning the various ways of managing CE practices by SMEs, which pertain to the identified pillars and the different stages of maturity and which can be investigated both at the macro (country) and meso (industry) levels and connected with commitment and support on the part of governments and institutions.

Additional contributions from scholars are also necessary to increase the awareness of SMEs concerning the path towards a circular vision of their future, which is not limited to single or market-pressured projects, as well as to increase the awareness of policy-makers concerning the fact that the effectiveness of their interventions and future key priorities depend on the adoption of a culture of sustainability and a global approach to circularity among SMEs.

## References

[CR1] Aghelie A (2017). Exploring drivers and barriers to sustainability green business practices within small medium sized enterprises: Primary findings. International Journal of Economics and Business.

[CR2] Almagtome A, Khaghaany M, Once S (2020). Corporate governance quality, stakeholders’ pressure, and sustainable development: An integrated approach. International Journal of Mathematical, Engineering and Management Sciences.

[CR3] Antony J, Kumar M, Labib A (2008). Gearing six sigma into UK manufacturing SMEs: Results from a pilot study. Journal of the Operational Research Society.

[CR4] Antony J, Vinodh S, Gijo EV (2016). Lean six sigma for small and medium sized enterprises: A practical guide.

[CR5] Avelino F, Rotmans J (2011). A dynamic conceptualization of power for sustainability research. Journal of Cleaner Production.

[CR6] Awan U, Sroufe R (2022). Sustainability in the circular economy: insights and dynamics of designing circular business models. Applied Science.

[CR7] Bag S, Pretorius JHC, Gupta S, Dwivedi YK (2021). Role of institutional pressures and resources in the adoption of big data analytics powered artificial intelligence, sustainable manufacturing practices and circular economy capabilities. Technological Forecasting and Social Change.

[CR8] Bassi F, Dias JG (2019). The use of circular economy practices in SMEs across the EU. Resources, Conservation & Recycling.

[CR9] Bassi F, Dias JG (2020). Sustainable development of small- and medium-sized enterprises in the European Union: A taxonomy of circular economy practices. Business Strategy and the Environment.

[CR10] Battaglia M, Bianchi L, Frey M, Iraldo F (2010). An Innovative model to promote CSR among SMEs operating in industrial clusters: Evidence from an EU project. Corporate Social Responsibility and Environmental Management.

[CR11] Bengtsson M (2016). How to plan and perform a qualitative study using content analysis. Nursing plus Open.

[CR12] Berkhout F, Smith A, Stirling A, Elzen B, Geels FW, Green K (2004). Socio-technical regimes and transition contexts. System innovation and the transition to sustainability.

[CR13] Bianchi R, Noci G (1998). ‘Greening’ SMEs’ competitiveness. Small Business Economics.

[CR14] Björklund M, Martinsen U, Abrahamsson M (2012). Performance measurements in the greening of supply chains. Supply Chain Management: An International Journal.

[CR15] Bocken NMP, de Pauw I, Bakker C, Van der Grinten B (2016). Product design and business model strategies for a circular economy. Journal of Industrial and Production Engineering.

[CR16] Bos-Brouwers H (2009). Corporate sustainability and innovation in SMEs: Evidence of themes and activities in practice. Business Strategy and the Environment.

[CR17] Brammer RG, Pavelin S (2008). Factors influencing the quality of corporate environmental disclosure. Business Strategy and the Environment.

[CR18] Braungart M, McDonough W, Bollinger A (2006). Cradle-to-cradle design: Creating healthy emissions—A strategy for eco-effective product and system design. Journal of Cleaner Production.

[CR19] Brendzel-Skowera K (2021). Circular economy business models in the SME sector. Sustainability.

[CR20] Brondoni SM (2008). Market-driven management, competitive space and global networks. Symphonya, Emerging Issues in Management.

[CR21] Brondoni SM (2020). Competitive circular economy management. The Mitsubishi corporation case. Symphonya, Emerging Issues in Management.

[CR22] Catanzaro M, Woods N, Catanzaro M (1988). Using qualitative analytical techniques. Nursing research: Theory and practice.

[CR23] Chen L, Hung P, Ma H (2020). Integrating circular business models and development tools in the circular economy transition process: A firm-level framework. Business Strategy and the Environment.

[CR24] Chizaryfard A, Trucco P, Nuur C (2020). The transformation to a circular economy: Framing an evolutionary view. Journal of Evolutionary Economics.

[CR25] Clark JH, Farmer TJ, Herrero-Davila L, Sherwood J (2016). Circular economy design considerations for research and process development in the chemical sciences. Green Chemistry.

[CR26] European Commission (EC) (2015). Closing the loop—An EU action plan for the circular economy COM/2015/0614 final. Retrieved 15 September 2021, from https://eur-lex.europa.eu/legal-content/EN/TXT/?uri=CELEX:52015DC0614.

[CR27] European Commission (EC) (2018). A sustainable bioeconomy for Europe: Strengthening the connection between economy, society and the environment: updated bioeconomy strategy. Retrieved 27 April 2022, from https://op.europa.eu/en/publication-detail/-/publication/edace3e3-e189-11e8-b690-01aa75ed71a1/language-en/format-PDF/source-149755478

[CR28] Cramer JM (2020). Implementing the circular economy in the Amsterdam metropolitan area: The interplay between market actors mediated by transition brokers. Business Strategy and the Environment.

[CR29] de Jesus A, Mendonça S (2018). Lost in transition? Drivers and barriers in the eco innovation road to the circular economy. Ecological Economics.

[CR30] de Sousa JABL (2019). Going in circles: New business models for efficiency and value. Journal of Business Strategy.

[CR31] Denzin NK, Lincoln YS, Denzin NK, Lincoln YS (2008). Introduction: The discipline and practice of qualitative research. Strategies of qualitative inquiry.

[CR32] Dolnicar S, Zare S (2020). COVID19 and Airbnb—disrupting the disruptor. Elsevier Public Health Emergency Collection.

[CR33] Domenech T, Bleischwitz R, Doranova A, Panayotopoulos D, Roman L (2019). Mapping industrial symbiosis development in Europe. Typologies of networks, characteristics, performance and contribution to the circular economy. Resources, Conservation and Recycling.

[CR34] Dubey R, Gunasekaran A, Childe SJ, Blome C, Papadopoulos T (2019). Big data and predictive analytics and manufacturing performance: Integrating institutional theory, resource-based view and big data culture. British Journal of Management.

[CR35] Circle Economy (2021). Circular jobs bulletins. Retrieved 12 July, 2021 from https://assets.website-files.com/5d26d80e8836af2d12ed1269/627925d40ee02fd76856c600_Circular%20Jobs%20Bulletin%202021.pdf

[CR36] Eikelenboom M, de Jong G (2021). The impact of managers and network interactions on the integration of circularity in business strategy. Organization & Environment.

[CR37] El Bilali H (2020). Transition heuristic frameworks in research on agro-food sustainability transitions. Environment, Development and Sustainability.

[CR38] Elia V, Gnoni MG, Tornese F (2017). Measuring circular economy strategies through index methods: A critical analysis. Journal of Cleaner Production.

[CR39] Ellen MacArthur Foundation (EMF) (2013). Towards the circular economy. Retrieved 2 August, 2021 from https://www.ellenmacarthurfoundation.org/assets/downloads/publications/Ellen-MacArthur-Foundation-Towards-the-Circular-Economy-vol.1.pdf

[CR40] Elo S, Kyngäs H (2008). The qualitative content analysis process. Journal of Advanced Nursing.

[CR41] Endrikat J, Guenther E, Hoppe H (2014). Making sense of conflicting empirical findings: A meta-analytic review of the relationship between corporate environmental and financial performance. European Management Journal.

[CR42] European Investment Bank (EIB) (2020). The EIB Circular Economy Guide. Supporting the circular transition. Retrieved 2 May 2022, https://www.eib.org/en/publications/the-eib-in-the-circular-economy-guide.

[CR43] European Commission (EC) (2020). An SME strategy for a sustainable and digital Europe. COM(2020) 103 final. Retrieved 5 September 2021, from https://eur-lex.europa.eu/legal-content/EN/TXT/HTML/?uri=CELEX:52020DC0103&from=EN.

[CR44] Eurostat (2020) SME Annual Report 2018–2019. Executive Summary. Retrieved 5 September 2021, https://ec.europa.eu/search/index.do?queryText=SMEs+employment&query_source=europa_default&filterSource=europa_default&swlang=en&more_options_language=en&more_options_f_formats=&more_options_date.

[CR45] Ferrucci L, Varaldo R (1996). The evolutionary nature of the firm within industrial districts. European Planning Studies.

[CR46] Flick U (2009). An introduction to qualitative research.

[CR47] Font X, Garay L, Jones S (2016). Sustainability motivations and practices in small tourism enterprises in European protected areas. Journal of Cleaner Production.

[CR48] Gallaud D, Laperche B (2016). Circular economy.

[CR49] Geissdoerfer MP, Pieroni MPP, Pigosso DCA, Soufani K (2020). Circular business models: A review. Journal of Cleaner Production.

[CR50] Geissdoerfer MP, Savaget P, Bocken NMP, Hultink EJ (2017). The circular economy—a new sustainability paradigm?. Journal of Cleaner Production.

[CR51] Geng Y, Doberstein B (2008). Developing the circular economy in China: Challenges and opportunities for achieving ‘leapfrog development’. The International Journal of Sustainable Development and World Ecology.

[CR52] Geng Y, Fu J, Sarkis J, Xue B (2012). Towards a national circular economy indicator system in China: An evaluation and critical analysis. Journal of Cleaner Production.

[CR53] Gennari F, Cassano R (2020). Circular economy and strategic risk. Symphonya, Emerging Issues in Management.

[CR54] Gennari F, Salvioni DM (2019). CSR committees on boards: the impact of the external country level factors. Journal of Management and Governance.

[CR55] Ghobadian A, Gallear DN (1996). Total quality management in SMEs. Omega.

[CR56] Girotra K, Netessine S (2011). How to build risk into your business model? Smart companies design their innovations around managing risks. Harvard Business Review.

[CR57] Glaser B (1978). Theoretical sensitivity.

[CR58] Golinelli, G. (2012). L’approccio sistemico (ASV) al governo dell’impresa. CEDAM.

[CR59] Green S, Thorogood N (2004). Qualitative methods for health research.

[CR60] Grin J, Rotmans J, Schot J, Loorbach D, Geels FW (2010). Transitions to sustainable development; new directions in the study of long term transformative change.

[CR61] Guidehouse Netherlands (2020). Market state and trends in renewable and low-carbon gases in Europe. Retrieved 30 September 2021, from https://www.consorzio.it/wp-content/uploads/2020/02/Gas-for-Climate-Market-State-and-Trends-report-2020.pdf

[CR62] Heat H, Cowley S (2004). Developing a grounded theory approach: A comparison of Glaser and Strauss. International Journal of Nursing Studies.

[CR63] Hennink M, Hutter I, Bailey A (2020). Qualitative research methods.

[CR64] Hernández-Chea R, Jain A, Bocken NMP, Gurtoo A (2021). The business model in sustainability transitions: A conceptualization. Sustainability.

[CR65] Hienerth C, Keinz P, Lettl C (2011). Exploring the nature and implementation process of user-centric business models. Long Range Planning.

[CR66] Holt DT, Vardaman JM (2013). Toward a comprehensive understanding of readiness for change: The case for an expanded conceptualization. Journal of Change Management.

[CR67] Holzer D, Rauter R, Fleiß E, Stern T (2021). Mind the gap: Towards a systematic circular economy encouragement of small and medium-sized companies. Journal of Cleaner Production.

[CR68] Homrich AS, Galvāo L, Abadia LG, Carvalho MM (2018). The circular economy umbrella: Trends and gaps on integrating pathways. Journal of Cleaner Production.

[CR69] Hoppe T, Kuokkanen A, Mikkilä M, Kahiluoto H, Kuisma M, Arentsen M, Linnanen L (2016). System merits or failures? Policies for transition to sustainable P and N systems in The Netherlands and Finland. Sustainability.

[CR70] Hörisch J, Freeman R, Schaltegger S (2014). Applying stakeholder theory in sustainability management: Links, similarities, dissimilarities, and conceptual framework. Organisation & Environment.

[CR71] Hsieh H, Shannon SE (2005). Three approaches to qualitative content analysis. Qualitative Health Research.

[CR157] International Energy Agency (2021) from https://www.iea.org/fuels-and-technologies/bioenergy

[CR72] Jabbour CJC (2010). Non-linear pathways of corporate environmental management: A survey of ISO 14001-certified companies in Brazil. Journal of Cleaner Production.

[CR73] Jackson M, Lederwasch A, Giurco D (2014). Transitions in theory and practice: Managing metals in the circular economy. Resources.

[CR74] Jakhar SK, Mangla SK, Luthra S, Kusi-Sarpong S (2019). When stakeholder pressure drives the circular economy. Measuring the mediating role of innovation capabilities. Management Decision.

[CR75] Jenkins HM (2004). A critique of conventional CSR theory: An SME perspective. Journal of General Management.

[CR76] Kazancoglu I, Kazancoglu Y, Kahraman A, Yarimoglu E, Soni G (2020). Investigating barriers to circular supply chain in the textile industry from Stakeholders’ perspective. International Journal of Logistics Research and Applications.

[CR77] Kemp R, Lehmann-Waffenschmidt M (2007). An example of a “Managed transition”: The transformation of the waste management subsystem in the Netherlands (1960–2000). Innovation towards sustainability: Conditions and consequences.

[CR78] Kirchherr J, Piscicelli L, Bour R, Kostense-Smit E, Muller J, Huibrechtse-Truijens A, Hekkert M (2018). M. Barriers to the circular economy: Evidence from the European Union (EU). Ecological Economics.

[CR79] Kirchherr J, Reike D, Hekkert M (2017). Conceptualizing the circular economy: An analysis of 114 definitions. Resources, Conservation and Recycling.

[CR80] Klewitz, J., & Hansen, E. G. (2011). Sustainability-oriented innovation in SMEs: A systematic literature review of existing practices and actors involved. In: Paper presented at the XXII International Society for Professional Innovation Management (ISPIM) Conference, Hamburg, Germany, June.

[CR81] Koe WL, Omar R, Sa’Ari JR (2015). Factors influencing propensity to sustainable entrepreneurship of SMEs in Malaysia. Procedia—Social and Behavioral Sciences.

[CR82] Korhonen J, Honkasalo A, Seppälä J (2018). Circular economy: The concept and its limitations. Ecological Economics.

[CR83] Korhonen J, Nuur C, Feldmann A, Birkie SE (2018). Circular economy as an essentially contested concept. Journal of Cleaner Production.

[CR84] Kracauer S (1952). The challenge of qualitative content analysis. The Public Opinion Quarterly.

[CR85] Kumar M, Khurram KK, Waddell D (2014). Status of quality management practices in manufacturing SMEs: A comparative study between Australia and the UK. International Journal of Production Research.

[CR86] Lessidrenska, T. (2019). SMEs and SDGs: Challenges and opportunities. OECD Development Matters. Retrieved 10 November 2021, from https://oecd-development-matters.org/2019/04/23/smes-and-sdgs-challenges-and-opportunities.

[CR87] Li K, Rollins J, Yan E (2018). Web of science use in published research and review papers 1997–2017: A selective, dynamic, cross-domain, content-based analysis. Scientometrics.

[CR88] Li S (2012). The research on quantitative evaluation of circular economy based on waste input-output analysis. Procedia Environmental Sciences.

[CR89] Litton J, Solomon G (2017). Technology, innovation, entrepreneurship and the small business—technology and innovation in small business. Journal of Small Business Management.

[CR90] Liu H, Yang H (2019). Managing network resource and organizational capabilities to create competitive advantage for SMEs in a volatile environment. Journal of Small Business Management.

[CR91] Loorbach D (2007). Transition management: New mode of governance for sustainable development.

[CR92] Loorbach D, Rotmans J (2010). The practice of transition management: Examples and lessons from four distinct cases. Futures.

[CR93] Loorbach D, Wijsman K (2013). Business transition management: Exploring a new role for business in sustainability transitions. Journal of Cleaner Production.

[CR94] Lozano R, Barreiro-Gen M (2021). Disrupting the brave new world: COVID-19 effects on organisations' sustainability efforts. Journal of Organizational Change Management.

[CR95] Markard J, Raven R, Truffer B (2012). Sustainability transitions: An emerging field of research and its prospects. Research Policy.

[CR96] Martin-Tapia I, Aragon-Correa JA, Senise-Barrio ME (2008). Being green and export intensity of SMEs: The moderating influence of perceived uncertainty. Ecological Economics.

[CR97] Matten D, Moon J, Habisch A, Jonker J, Wagner M, Schmidpeter R (2004). A conceptual framework for understanding CSR. Corporate social responsibility across Europe.

[CR98] Maxwell JA (2005). Qualitative research design: An interactive approach.

[CR99] McIntyre K, Ortiz JA, Clift R, Druckman A (2016). Multinational corporations and the circular economy: How Hewlett packard scales innovation and technology in its global supply chain. Taking stock of industrial ecology.

[CR100] Mendoza JMF, Sharmina M, Gallego-Schmid A, Heyes G, Azapagic A (2017). Integrating back casting and eco-design for the circular economy: The BECE framework. Journal of Industrial Ecology.

[CR101] Miles MB, Huberman AM (1994). Qualitative data analysis: An expanded sourcebook.

[CR102] Moher D, Liberati A, Tetzlaff J, Altman DG, Group P (2009). Preferred reporting items for systematic reviews and meta-analyses: The PRISMA statement. Open Medicine.

[CR103] Morgan J, Mitchell P (2015). Employment and the circular economy. Job creation in a more resource efficient Britain. Green Alliance.

[CR104] Morse MM, Richards L (2002). README FIRST for a user's guide to qualitative methods.

[CR105] Munn Z, Stern C, Aromataris E, Lockwood C, Jordan Z (2018). What kind of systematic review should I conduct? A proposed typology and guidance for systematic reviewers in the medical and health sciences. BMC Medical Research Methodology.

[CR106] Musa H, Chinniah M (2016). Malaysian SMEs development: Future and challenges on going green. Procedia Social and Behavioral Sciences.

[CR107] Noci G, Verganti R (1999). Managing 'green' product innovation in small firms. R&D Management.

[CR108] Nußholz J (2017). Circular business models: Defining a concept and framing an emerging research field. Sustainability.

[CR109] Oncioiu I, Căpuşneanu S, Türkeș MC, Topor DI, Constantin DMO, Marin-Pantelescu A, Hint MS (2018). The sustainability of Romanian SMEs and their involvement in the circular economy. Sustainability.

[CR110] Ormazabal M, Prieto Sandoval V, Puga-Leal R, Jaca C (2018). Circular economy in Spanish SMEs: Challenges and opportunities. Journal of Cleaner Production.

[CR111] Ormazabal M, Sarriegi JM, Barkemeyer R, Viles E, McAnulla F (2015). Evolutionary pathways of environmental management in UK companies. Corporate Social Responsibility and Environmental Management.

[CR112] Pauli G (2010). The blue economy: 10 Years, 100 innovations, 100 million jobs.

[CR113] Pearce DW, Turner RK (1990). Economics of natural resources and the environment.

[CR114] Perrini F, Russo A, Tencati A (2007). CSR strategies of SMEs and large firms. Evidence from Italy. Journal of Business Ethics.

[CR115] Pheifer, A. G. (2017). Whitepaper. Barriers and enablers to circular business models. Retrieved 5 August 2021, from https://www.circulairondernemen.nl/uploads/4f4995c266e00bee8fdb8fb34fbc5c15.pdf

[CR116] Pranckutė R (2021). Web of science (WoS) and scopus: The Titans of bibliographic information in today’s academic world. Publications.

[CR117] Prieto-Sandoval V, Jaca C, Ormazabal M (2018). Towards a consensus on the circular economy. Journal of Cleaner Production.

[CR119] Rizos V, Behrens A, Van der Gaast W, Hofman E, Ioannou A, Kafyeke T, Flamos A, Rinaldi R, Papadelis S, Hirschnitz-Garbers M, Topi C (2016). Implementation of circular economy business models by small and medium-sized enterprises (SMEs): Barriers and enablers. Sustainability.

[CR118] Rizos, V., Tuokko, K., & Behrens, A. (2017). The circular economy a review of definitions, processes and impacts. CEPS Research Report No. 2017/09, Brussels

[CR120] Robinson J, Barron A, Pottinger L, Barron A, Browne AL, Ehgartner U, Hall SM, Pottinger L, Ritson J (2021). Open interviews. Methods for change: Impactful social science methodologies for 21st century problems.

[CR121] Rodgers C (2010). Sustainable entrepreneurship in SMEs: A case study analysis. Corporate Social Responsibility and Environmental Management.

[CR124] Rotmans J, Loorbach D (2009). Complexity and transition management. Journal of Industrial Ecology.

[CR122] Rotmans, J., Loorbach, D., & Kemp, R. (2007). Transition Management: Its origin, evolution and critique. Workshop on Politics and governance in sustainable socio-technical transitions, Schloss Blankensee, Berlin, Germany

[CR123] Rotmans J, Kemp R, Van Asselt M (2001). More evolution than revolution: Transition management in public policy. Foresight.

[CR125] Russo A, Tencati A (2009). A. Formal vs. informal CSR strategies: Evidence from Italian micro, small, medium-sized, and large firms. Journal of Business Ethics.

[CR126] Saidani M, Yannou B, Leroy Y, Cluzel F (2017). How to assess product performance in the circular economy? Proposed requirements for the design of a circularity measurement framework. Recycling.

[CR128] Salvioni DM, Almici A (2020). Circular economy and stakeholder engagement strategy. Symphonya, Emerging Issues in Management.

[CR129] Salvioni DM, Bosetti L, Fornasari T (2022). Implementing and monitoring circular business models: An analysis of Italian SMEs. Sustainability.

[CR130] Salvioni DM, Gennari F (2017). CSR, sustainable value creation and shareholder relations. Symphonya, Emerging Issues in Management.

[CR127] Salvioni, D. M., Gennari, F., & Cassano R. (2022b). Risk management in circular economy strategies. In: V. Dell’Atti, A. L. Muserra, S. Marasca, & R. Lombardi (Eds.). Dalla Crisi allo Sviluppo Sostenibile. Franco Angeli.

[CR131] Sargeant JM, Rajic A, Read S, Ohlsson A (2006). The process of systematic review and its application in agri-food public-health. Preventive Veterinary Medicine.

[CR132] Scipioni S, Russ M, Niccolini F (2021). From barriers to enablers: The role of organizational learning in transitioning SMEs into the circular economy. Sustainability.

[CR133] Sharma NK, Govindan K, Lai KK, Chen WK, Kumar V (2020). The transition from linear economy to circular economy for sustainability among SMEs: A study on prospects, impediments, and prerequisites. Business Strategy and the Environment.

[CR134] Shields J, Shelleman JM (2015). Integrating sustainability into SME strategy. Journal of Small Business Strategy.

[CR135] Siegel R, Antony J, Garza-Reyes JA, Cherrafi A, Lameijer B (2019). Integrated green lean approach and sustainability for SMEs: From literature review to a conceptual framework. Journal of Cleaner Production.

[CR136] Sofaer S (1999). Qualitative methods: What are they and why use them?. Health Services Research.

[CR137] Srisathan WA, Naruetharadhol P (2022). A COVID-19 disruption: The great acceleration of digitally planned and transformed behaviors in Thailand. Technology in Society.

[CR138] Suchek N, Ferreira JJ, Fernandes PO (2022). A review of the entrepreneurship and circular economy research: State of the art and future directions. Business Strategy and the Environment.

[CR139] Svensson N, Funck EK (2019). Management control in circular economy. Exploring and theorizing the adaptation of management control to circular business models. Journal of Cleaner Production.

[CR140] Tagliafierro N (2020). The circular economy at Enel X. Symphonya, Emerging Issues in Management.

[CR141] Tang M, Liao H (2021). Multi-attribute large-scale group decision making with data mining and subgroup leaders: An application to the development of the circular economy. Technological Forecasting and Social Change.

[CR142] The Globalization Council (2009). The role of SMEs and entrepreneurship in a globalized economy. Retrieved 2 August 2021, from https://www.government.se/49b731/contentassets/8efd3c3a4c844f88883513fa451760bd/the-role-of-smes-and-entrepreneurship-in-a-globalised-economy

[CR143] Thorley J, Garza-Reyes JA, Anosike A (2022). Circular economy: A conceptual model to measure readiness for manufacturing SMEs. Benchmarking: an International Journal.

[CR144] Tilley F (2000). Small firm environmental ethics: How deep do they go?. Business Ethics.

[CR145] Truant E, Culasso F, Argento D (2019). Disclosing strategies and business models in the integrated report. Symphonya, Emerging Issues in Management.

[CR146] Tura N, Hanski J, Ahola T, Ståhle M, Piiparinen S, Valkokari P (2019). Unlocking circular business: A framework of barriers and drivers. Journal of Cleaner Production.

[CR147] Van Bakel J, Loorbach D, Whiteman G, Rotmans J (2009). Business strategies for transitions towards sustainable systems. Business Strategy and the Environment.

[CR148] Vovchenko NG, Epifanova TV, Zolochevskaya EY, Litvinova SA (2020). The culture of responsible production and consumption as a foundation of the circular economy in countries of Western Europe. Circular economy in developed and developing countries: Perspective, methods and examples.

[CR149] Webster K (2015). The circular economy: A wealth of flows.

[CR150] Witjes S, Lozano R (2016). Towards a more circular economy: Proposing a framework linking sustainable public procurement and sustainable business models. Resources, Conservation and Recycling.

[CR151] Yadow N, Gupta K, Rani L, Rawat D (2018). Drivers of sustainability practices and SMEs: A systematic literature review. European Journal of Sustainable Development.

[CR152] Yin RK (2003). Case study research: Design and methods.

[CR153] Yusof SM, Aspinwall E (2000). A conceptual framework for TQM implementation for SMEs. TQM Magazine.

[CR154] Zhang A, Venkatesh VG, Liu Y, Wan M, Qu T, Huisingh D (2019). Barriers to smart waste management for a circular economy in China. Journal of Cleaner Production.

[CR155] Zhu B, Nguyen M, Sarm Siri N, Malik A (2022). Towards a transformative model of circular economy for SMEs. Journal of Business Research.

